# OA05 Cycling: a tool for cardiovascular exercise in patients with established ankylosing spondyloarthritis

**DOI:** 10.1093/rap/rkad070.005

**Published:** 2023-09-27

**Authors:** Vera Saulite, Fatima Salih, Sandeep Dahiya

**Affiliations:** North West Anglia NHS Foundation Trust, Huntingdon, United Kingdom; North West Anglia NHS Foundation Trust, Peterborough, United Kingdom; North West Anglia NHS Foundation Trust, Peterborough, United Kingdom

## Abstract

**Introduction:**

Ankylosing spondyloarthritis (AS) is a chronic inflammatory disease that can lead to irreversible changes in spine and sacroiliac joints, affecting spinal mobility adversely, leading to disability and severe functional impairment. Use of biological treatments in recent years has led to significant improvement in ability to control disease activity. Exercise remains an essential part of managing inflammatory arthritis such as AS. We present a case of long-standing AS which was in remission according to clinical calculators. However, it was the introduction of regular aerobic exercise that has greatly improved the patient's remaining musculoskeletal symptoms, sense of wellbeing, and energy levels.

**Case description:**

A 64-year-old man was diagnosed with AS in the 1980s at age 27. He was managed with treatments available at the time such as nonsteroidal anti-inflammatory drugs and physiotherapy, including hydrotherapy, with relatively poor response, leading to gradual progression of disease and limitation in his spinal mobility and much stooped posture. He started adalimumab in 2008, leading to significant improvement in his disease activity. Despite achieving remission, he felt tired and although he was relatively pain free, it did not translate into an improved sense of wellbeing. He felt generally unfit and developed hypertension, requiring three antihypertensive medications and put on a few pounds.

As part of his rheumatology consultation in 2012, it was suggested that cardiovascular exercise should help improve his fitness, hypertension, tiredness and sense of wellbeing. Due to spinal limitation, swimming and running were not suitable options. He therefore tried indoor rowing, which unfortunately led to worsening pain in his lower back within days. Instead of giving up, he decided to give cycling a trial. After initial discomfort, he was able to cycle regularly and started to feel the benefit within weeks. He then got involved a bit more and joined a local cycling club and was doing 10,000 km per year. He completed the Paris to London cycle ride for a charity. Within the first year of taking up regular cycling, he lost a couple of stone in weight, and now requires only one antihypertensive.

Although his disease went into remission fairly soon after taking adalimumab and he became pain free, it was only with cardiovascular exercise he felt the real sense of wellbeing. He thinks that cycling has made a significant change in physical and psychological health.

**Discussion:**

A recent meta-analysis of 14 randomised controlled studies, including 1,579 participants, demonstrated moderate‐to-low quality evidence that exercise programmes slightly improve function, may reduce pain, and probably slightly reduce global patient assessment of disease activity when compared with no intervention. This study also showed that exercise programmes probably have minimal or no effect on function or pain when compared with usual care, and may have little or no effect on reducing patient assessment of disease activity. There is still no sufficient evidence to determine whether exercise programmes improve spinal mobility, reduce fatigue, or induce adverse effects.

Nevertheless, exercise programmes are given a prominent role in treatment of AS. The evidence is primarily focused on mobility exercise rather than strengthening, balance or cardio-respiratory exercise. There is also an unmet need for information about exercise planning, frequency, intensity and duration or adherence to recommended programmes.

Physical therapy with supervised exercises has been recommended and is thought to be better than home exercises. In one study, a supervised exercise program arm where exercise consisted of aerobic (treadmill), resistance and stretching exercises for 1 hour a day, five days a week for three weeks has shown to improve BASDAI and BASFAI. However, this result was not statistically significant when compared to the control group with home-based exercise. It also showed that secretion of the antioxidant responsible for inflammation decreases with intensive cardiovascular exercise. A systematic review of available evidence has suggested benefits of personalised exercise regimen in AS patients. Our case illustrates that regular cardiovascular exercise can improve the musculoskeletal, cardiovascular and psychological health in chronic inflammatory conditions like AS which are otherwise to be considered in remission as per available disease activity measuring tools.

**Key learning points:**

There is evidence for the role of exercise in the management of AS which includes stretching, core work and posture advice, as well as cardiovascular work. The role of cardiovascular exercise in all forms of inflammatory arthritis is well established and is not only safe but also has been shown to have beneficial effects on cardiovascular risk, bone health, psychological health.

This case highlights the importance of devising a personalised exercise programme for individual patients that takes into account any previous experience, physical limitations, lifestyle and any other barriers. We have been advising patients with established AS to try cycling or exercise bikes as one of the tools for achieving cardiovascular workout in a safe and effective manner and have seen several patients finding it useful. There is limited literature about use of cardiovascular exercise in AS, and no specific studies looking at cycling. There are, however, multiple individual patient stories online describing the benefits of cycling in AS. We feel the effectiveness of cycling can be further explored by setting up observational studies.

